# A change in temporal muscle thickness is correlated with past energy adequacy in bedridden older adults: a prospective cohort study

**DOI:** 10.1186/s12877-021-02086-0

**Published:** 2021-03-15

**Authors:** Yoko Hasegawa, Mikako Yoshida, Aya Sato, Yumiko Fujimoto, Takeo Minematsu, Junko Sugama, Hiromi Sanada

**Affiliations:** 1 Department of Gerontological Nursing/Wound Care Management, Graduate School of Medicine, The University of Tokyo, 7-3-1 Hongo, Bunkyo-ku, Tokyo, 113-0033 Japan; 2 Clinical Nutrition Center, The University of Tokyo Hospital, 7-3-1 Hongo, Bunkyo-ku, Tokyo, 113-8655 Japan; 3 Department of Women’s Health Nursing & Midwifery, Tohoku University Graduate School of Medicine, 2-1 Seiryoumachi, Aoba-ku, Sendai city, Miyagi 980-8575 Japan; 4 Department of Gerontological Nursing, Kawasaki City College of Nursing, 4-30-1 Ogura, Saiwai-ku, Kawasaki city, Kanagawa 212-0054 Japan; 5 Department of Gerontological Nursing, School of Health and Nursing Science, Wakayama Medical University, 580 Mikazura, Wakayama city, Wakayama 641-0011 Japan; 6 Department of Skincare Science, Graduate School of Medicine, The University of Tokyo, 7-3-1 Hongo, Bunkyo-ku, Tokyo, 113-0033 Japan; 7 Division of Care Innovation, Global Nursing Research Center, Graduate School of Medicine, The University of Tokyo, 7-3-1 Hongo, Bunkyo-ku, Tokyo, 113-0033 Japan; 8 Institute for Frontier Science Initiative, Kanazawa University, 5-11-80 Kodatsuno, Kanazawa city, Ishikawa 920-0942 Japan

**Keywords:** Energy adequacy, Malnutrition, Nutritional monitoring, Temporal muscle, Ultrasonography

## Abstract

**Background:**

Energy inadequacy has a great impact on health outcomes in older adult patients; however, it is difficult to evaluate energy adequacy in these patients, especially in home-care settings. We recently reported that temporal muscle thickness can be an indicator of nutritional status. The present study aims to examine whether a change in temporal muscle thickness is directly correlated with energy adequacy and, if so, to determine the cutoff value of a change in temporal muscle thickness to detect energy inadequacy.

**Methods:**

A prospective cohort study was conducted from September 2015 to June 2016 in two hospitals in Japan, and included bedridden older adult patients aged ≥65 years. Temporal muscle thickness was measured using ultrasonography. Energy intake was estimated by photographic diet records. Total energy expenditure (TEE) was estimated by multiplying basal energy expenditure calculated using the Harris– Benedict equation by activity and stress factors. Energy adequacy was then calculated by dividing TEE by energy intake. Pearson’s correlation coefficient was used to examine the relationship between percentage change in temporal muscle thickness and energy adequacy. Multiple logistic regression analysis was conducted to determine the direct relationship between percentage change in temporal muscle thickness and moderate energy inadequacy (energy adequacy< 75%). Receiver operating characteristic (ROC) analysis was performed to determine the cutoff point for percentage change in temporal muscle thickness to detect moderate energy inadequacy.

**Results:**

Forty-eight patients were analyzed (mean age 84.4 ± 7.8 years; 54.2% were women). The percentage change in muscle thickness was significantly correlated with energy adequacy (*r* = 0.733, *p* < 0.001). ROC analysis identified a percentage change in temporal muscle thickness of − 3.6% as the optimal cutoff point for detecting moderate energy inadequacy. Percentage change in muscle thickness was independently correlated with energy inadequacy after adjusting for age, sex, and masticatory status (AOR 0.281, 95% CI 0.125–0.635).

**Conclusions:**

Changes in temporal muscle thickness are directly correlated with energy adequacy and can indicate moderate energy inadequacy in bedridden older adults. These results suggest the assessment of changes in temporal muscle thickness could be useful for guiding nutritional care in older adult patients in home-care settings.

## Background

Malnutrition in older adults receiving home care is a problem worldwide. The prevalence and risk of malnutrition in home-care settings have been reported to be 8.9–24.6% and 51.2–67.4%, respectively [1–5]. Malnutrition leads to a decline in the activities of daily living (ADL) [2, 4] and an increase in rates of infection and mortality [1, 5]. Hence, nutritional care that prevents or ameliorates malnutrition is crucial in a home-care setting.

Nutritional care consists of the following four steps: nutritional assessment, nutritional diagnosis, nutritional intervention, and nutritional monitoring [6]. Nutritional monitoring is essential for effective nutritional care because it evaluates the effectiveness of any nutritional interventions provided. In current clinical practice of nutritional management in home-care settings in Japan, nutritional monitoring is routinely performed approximately every 4 weeks, according to the medical insurance system.

One of the most important indicators used in nutritional monitoring is energy adequacy. Energy adequacy has a great impact on health outcomes in older adult patients. Moderate energy inadequacy (consuming 50–74% of the recommended requirement) in older adult patients can be an independent risk factor for various life-threatening complications, such as a dramatic deterioration in a patient’s clinical status or the need to transfer to an intensive care unit for monitoring and treatment [7]. Furthermore, severe energy inadequacy (consuming less than 50% of the recommended requirement) is a risk factor for inpatient mortality, functional dependency at discharge [8], and prolonged length of hospital stay [9]. Therefore, it is important to routinely monitor patients for energy inadequacy. However, the assessment of energy adequacy is often not feasible, especially in older adults who have impaired cognitive function, because they cannot recall their usual food intake nor keep a food diary. It is desirable to develop an objective method for nutritional monitoring of energy adequacy that is applicable for these older adult patients.

We have previously demonstrated that temporal muscle thickness can be an indicator of nutritional status in older adults [10]. Muscle mass changes in accordance with an imbalance of energy intake [11]; therefore, we speculated that assessing changes in temporal muscle thickness would be useful for nutritional monitoring of energy adequacy.

The aims of this study were to: 1) examine whether a change in temporal muscle thickness is significantly correlated with energy adequacy in bedridden older adults; 2) determine the cutoff value for percentage change in temporal muscle thickness to detect moderate energy inadequacy; and 3) confirm whether any change in temporal muscle thickness can be a nutritional indicator to detect past energy inadequacy independently of the possible cofounders, such as age, sex, and masticatory status.

## Methods

### Study design

A prospective cohort study was conducted from September 2015 to June 2016. The protocol was approved by the Research Ethics Committee of the Graduate School of Medicine, The University of Tokyo, and the board of the participating facility. Written informed consent was obtained from all participants or their family members.

### Participants

Older adult patients with impaired physical function were recruited from two facilities: a long-term care hospital and the rehabilitation unit of a general hospital in Kanazawa city, Ishikawa, Japan. Inclusion criteria were patients who were aged 65 years or older and who were in a bedridden state. A bedridden state was determined according to the criteria of the Japanese Ministry of Health, Labour and Welfare, which classify a patient’s level of dependency into one of three categories: independent, semi-bedridden, and bedridden. Exclusion criteria were as follows: history of wound, skin lesion, surgery, and/or radiation therapy in the temporal region, or history of muscular dystrophy; planned discharge within the next 4 weeks; and individuals with a terminal illness who were expected to be deceased within 4 weeks.

The sample size was set to 58. Based on our previous cross-sectional study in which we showed a significant correlation between temporal muscle thickness and daily energy intake (*r* = 0.439, *p* < 0.001; unpublished data), the expected effect size of correlation between a percentage change in temporal muscle thickness and energy adequacy was estimated as *r* = 0.45. The two-sided α was set to 0.05 and the β was set to 0.10. An anticipated dropout rate of 20% was added to the estimated sample size of 48.

### Procedure

After obtaining their informed consent, the researcher collected data regarding the basic characteristics of participants through a review of their medical charts. Then the researcher performed anthropometric measurements and ultrasonography of the temporal muscle at the baseline (0 weeks) and the endpoint (4 weeks). Baseline blood tests were performed within 7 days of the anthropometric measurements and ultrasonography of the temporal muscle at baseline. Energy intake was examined for 2 days during each week of the observation period, and the averaged values of each week were used for the analysis.

### Nutritional status

Baseline nutritional status was assessed using a Mini Nutritional Assessment short form (MNA-SF) [12], serum prealbumin level, and detailed anthropometry, including body mass index (BMI), arm circumference (AC), arm muscle circumference (AMC), and calf circumference (CC). Detailed procedures of the blood tests and anthropometric measurements are described elsewhere [10].

### Temporal muscle thickness

Ultrasonographic images were obtained using a portable ultrasonographic machine (M-Turbo; SonoSite, Bothwell, WA) with a high-frequency linear transducer (L25x/13–6 MHz; SonoSite), as previously reported [10]. The depth was kept at 3.7 cm. The gain and dynamic range were automatically adjusted to an appropriate level for each measurement and maintained at the same levels throughout the study.

Ultrasonography was performed with participants in the lateral position. The temporal muscle was scanned at 4.0 cm from the eyelid and 2.0 cm above the reference line, which was the horizontal line connecting the corner of the eyelid to the upper edge of the external auditory canal. The center of the transducer was placed transversally at this measurement site. A sufficient quantity of ultrasonographic gel was applied to a patient’s skin to minimize the influence of pressure when applying the transducer.

**Fig. 1:**
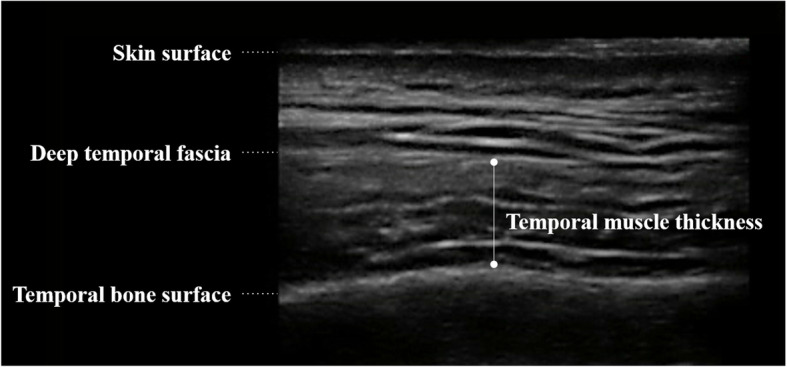
Ultrasonographic image of temporal muscle. Muscle thickness was defined as the distance between deep temporal fascia to the temporal bone surface. The exact location of measurement was defined upon agreement by three nursing researchers as the highest point in the skull surface in each image

Temporal muscle thickness was measured from the ultrasonographic images using ImageJ (National Institutes of Health, Bethesda, MD). Temporal muscle thickness was defined as the distance from the deep temporal fascia to the temporal bone surface (Fig. 1). The exact location of measurement was defined upon agreement by three nursing researchers as the highest point of the skull surface in each image.

### Energy intake

Energy intake was surveyed using the digital photographic method [13–15]. All meals, snacks, and beverages were recorded by digital photographs taken by the investigator. Photographs were taken directly above a meal with a reference scale placed by a plate before and after eating to record the amount of food served and the amount not eaten. Beverages and nutritional supplements were weighed before and after consumption, to the nearest 0.1 g, using digital scales (KD-180, TANITA, Tokyo, Japan). The energy content of each hospital meal was calculated by the food service dietitian at each facility. The investigator estimated the portion size consumed in each meal to the nearest 5%. Energy intake was then estimated by multiplying the energy content of each meal and the percentage of the meal consumed.

### Energy adequacy

Basal energy expenditure (BEE) was estimated using the Harris–Benedict (HB) equation [16]. Total energy expenditure (TEE) was estimated by multiplying BEE by a stress factor and an activity factor.

The stress factor was determined as follows, based on previous reports [17–22]: 1.3 (infection, or fever ≥37 degrees for ≥6 days or ≥ 38 degrees for ≥4 days over 4 weeks); 1.2 (cancer, pancreatic disease, pressure ulcer, post-fracture, or fever ≥37 degrees for ≥3 days or ≥ 38 degrees for ≥2 days over 4 weeks); 1.1 (chronic obstructive pulmonary disease, liver disease, sub-acute state of post-stroke); 0.9 (chronic state of post-stroke with enteral feeding and bedridden); and 1.0 (none of the above).

The activity factor was also determined based on previous reports [23–26]: 1.0 (bedridden with poor consciousness and unable to turn by oneself); 1.1 (bedridden with good consciousness but unable to turn over by oneself); 1.2 (able to turn over by oneself but limited to bed rest); 1.3 (some activity outside of bed, e.g., transferring to a wheelchair or short periods of indoor walking); 1.4 (aggressive rehabilitation for 20 to 40 min per day); 1.5 (aggressive rehabilitation for 40 to 60 min per day); 1.6 (aggressive rehabilitation for 60 to 80 min per day); 1.7 (aggressive rehabilitation for ≥90 min per day). The definition of the activity factor was reviewed and confirmed by an experienced physical therapist. Energy adequacy (%) was calculated by dividing TEE (kcal/day) by daily energy intake (kcal/day) and then multiplying this value by 100. Energy adequacies of less than 75% and less than 50% were determined to be moderate and severe energy inadequacy, respectively.

### Masticatory status

Masticatory status was defined and categorized into three groups according to a patient’s dietary texture: a regular to soft diet was categorized as “frequent mastication”; a minced or pureed diet was categorized as “a little mastication”; and those who were on either enteral or parenteral nutrition and not consuming any food orally were categorized as “no mastication”.

### Statistical analysis

Percentage change in temporal muscle thickness was calculated by dividing the change in temporal muscle thickness (baseline thickness subtracted from endpoint thickness) by its baseline value. Descriptive data were presented as means with standard deviation (SD). Correlations between a percentage change in temporal muscle thickness with energy adequacy were tested using Pearson’s correlation coefficient. Receiver operating characteristic (ROC) curve analysis was conducted to identify a cutoff value to detect energy inadequacy. To examine any direct relationships between percentage change in muscle thickness and energy adequacy, a multiple logistic regression analysis was conducted using moderate energy inadequacy as a dependent variable. Theoretically relevant factors chosen as independent variables were age, sex, masticatory status, and percentage change in temporal muscle thickness. Independent variables were assessed for multicollinearity and then simultaneously entered. All analyses were conducted using SPSS version 23.0 (IBM Corp., Armonk, NY). Statistical significance was set at *p* < 0.05.

## Results

We recruited 69 patients, of whom 6 patients declined to join this study. Written informed consent was obtained from 63 participants, 61 of whom completed the survey. Two patients dropped out, one due to withdrawal of consent (*n* = 1) and one who was discharged to go home (*n* = 1), a dropout rate of 3.2%. The ultrasonographic images obtained were examined by the investigator and two nursing researchers with experience in ultrasonography, to determine whether the image quality was sufficient to measure muscle thickness. Ultrasonographic images where the three assessors agreed the image quality was acceptable were selected for analysis. Thirteen participants (21.3%) were excluded from the analysis due to poor image quality. The average age of participants who dropped out or were excluded was 87.6 ± 6.7 years; ten (66.7%) were women. There were no significant differences in the basic characteristics between participants who completed the study and those who dropped out or were excluded from the analysis.

Ultimately, 48 participants were analyzed; their mean age was 84.4 ± 7.8 and 26 (54.2%) of them were women (Table 1). The most frequent past medical history was cerebrovascular disease (*n* = 20, 41.7%). There were 16 participants (33.3%) who were on either enteral or parenteral nutrition and not consuming any food orally. Temporal muscle thickness was 3.6 ± 0.9 mm at baseline and 3.5 ± 1.0 mm at 4 weeks. The difference in muscle thickness was − 0.1 ± 0.7 mm, and the percentage change in temporal muscle thickness was − 1.9 ± 21.8%. The mean BEE and TEE were 885 ± 118 kcal/day and 1248 ± 348 kcal/day, respectively. The mean activity and stress factors were 1.3 ± 0.2 and 1.1 ± 0.1, respectively. The mean energy adequacy was 92.2 ± 28.1%.

**Table 1:**
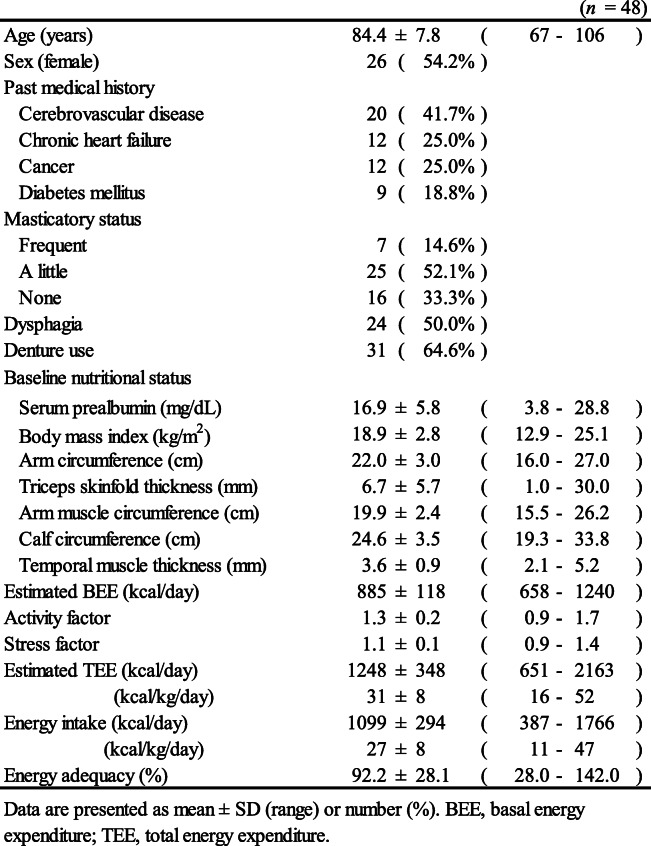
Patient characteristics

**Fig. 2:**
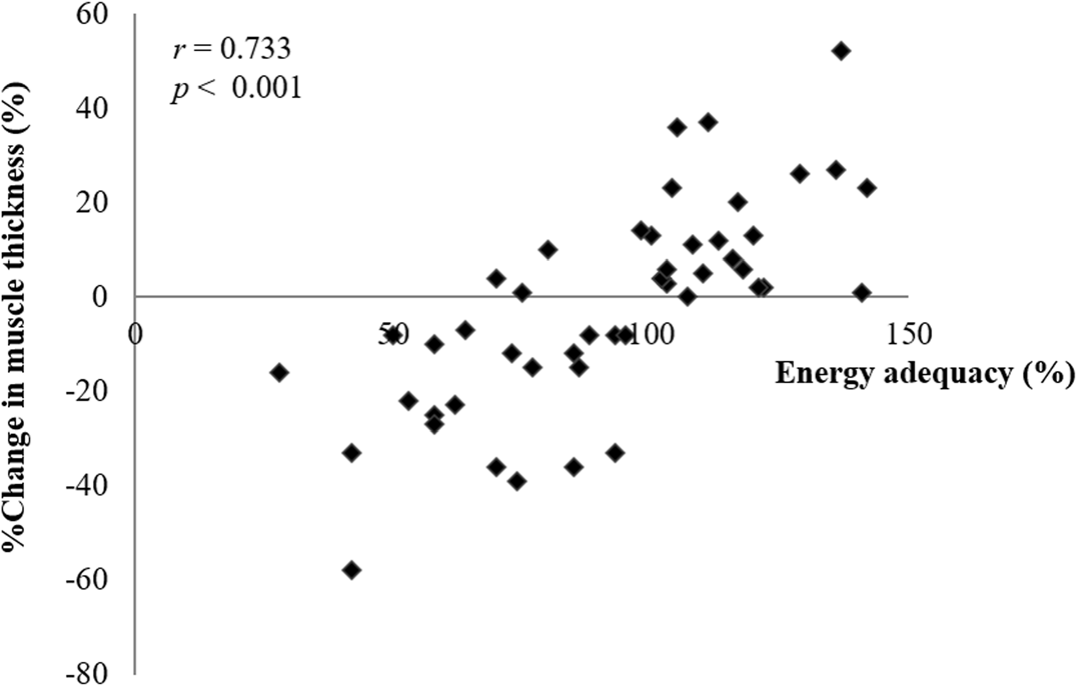
Correlation of percentage changes in muscle thickness with energy adequacy (*n* = 48). The percentage change in muscle thickness was calculated by dividing a change in temporal muscle thickness over 4 weeks by baseline thickness

Participants with higher energy adequacy had greater degrees of percentage change in temporal muscle thickness (*r* = 0.733, *p* < 0.001) (Fig. 2). This significant correlation was confirmed both in males (*r* = 0.763, *p* < 0.001) and females (*r* = 0.702, *p* < 0.001). Among those who lacked any masticatory activity (*n* = 16), the percentage change in temporal muscle thickness was also significantly correlated with past energy adequacy (*r* = 0.790, *p* < 0.001).

**Fig. 3:**
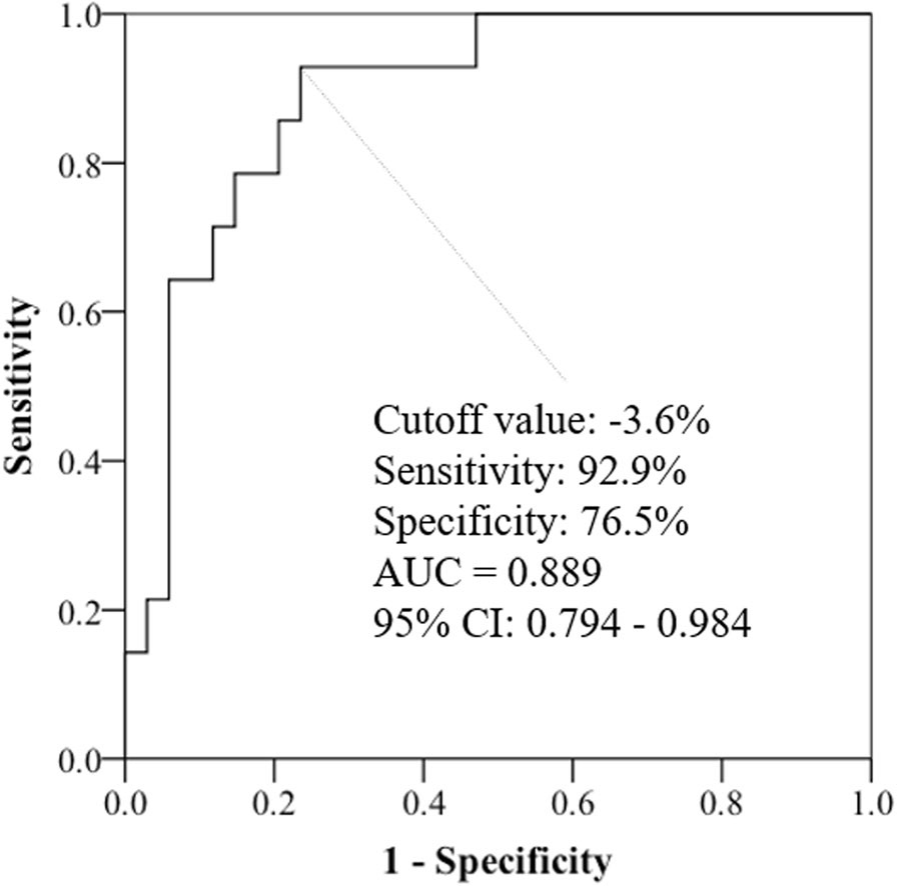
Receiver operating characteristic (ROC) curve analysis (*n* = 48). ROC curve analysis was conducted to determine the appropriate cutoff point of the percentage change in muscle thickness to detect energy inadequacy (Energy intake / TEE < 75%). These ROC curves were constructed by plotting sensitivity by (1 - specificity) for each value of daily energy intake. AUC, area under the curve; CI, confidential interval; TEE, total energy expenditure

The results of the ROC analysis revealed an optimal cutoff value of − 3.6% for detecting moderate energy inadequacy, with a sensitivity and specificity of 92.9 and 76.5%, respectively (Fig. 3). ROC analysis could not be conducted to determine a cutoff value for detecting severe energy inadequacy as there were only three participants with severe inadequate energy intake.

Consequently, multiple logistic regression analysis was performed to confirm whether the percentage change in muscle thickness was significantly related to energy inadequacy after adjusting for masticatory status, age, and sex (Table 2). The percentage change in muscle thickness (unit: 10%) was found to be independently significantly related to moderate energy after adjustments were made for masticatory status, age, and sex (adjusted odds ratio (AOR): 0.281, 95% CI: 0.125–0.635).

**Table 2:** Multiple logistic analysis of factors affecting moderate energy inadequacy (adequacy < 75%)

		(*n* = 48)									
		COR	*p*	95%CI			AOR	*p*	95%CI		
%Change in temporal muscle thickness (%)		0.364	0.001	0.201	–	0.659	0.281	0.002	0.125	–	0.635
Age		0.999	0.973	0.921	–	1.082	1.003	0.960	0.894	–	1.125
Sex (Female)		1.267	0.710	0.364	–	4.409	6.024	0.118	0.632	–	57.401
Masticatory status	Frequent	Ref					Ref				
	A little	2.250	0.397	0.345	–	14.694	2.033	0.634	0.109	–	37.799
	None	1.167	0.833	0.279	–	4.871	2.164	0.446	0.297	–	15.741

Hosmer-Lemeshow test, *p* = 0.552. Percentage of correct classification, 87.5%

Abbreviations: *COR* crude odds ratio, *AOR* adjusted odds ratio, *CI* confidential interval

Independent variables: age, sex, masticatory status, and percentage change in temporal muscle thickness

## Discussion

This study revealed for the first time that percentage change in temporal muscle thickness is significantly correlated with energy adequacy and that a percentage change in temporal muscle thickness can be used to detect moderate energy inadequacy. These results suggest that the assessment of percentage changes in temporal muscle thickness could be useful for nutritional monitoring. The cutoff value of the percentage change in temporal muscle thickness was − 3.6% for moderate energy inadequacy (adequacy < 75%).

A percentage change in temporal muscle thickness was significantly correlated with moderate energy inadequacy after adjustments for age, sex, and masticatory status. This result suggests that a percentage change in temporal muscle thickness can be used as a nutritional monitoring indicator in bedridden older adults of varying masticatory status, age, and sex. Since energy intake in this study was significantly correlated with protein intake (*r* = 0.914, *p* < 0.001), the decreased thickness in temporal muscle could be a reflection of the inadequacy of both energy and protein intake. Muscle mass can be reduced when the intake of energy and protein is inadequate, first because protein cannot be utilized efficiently to maintain muscle mass when energy intake is inadequate, and second because muscle catabolism can be induced when protein intake is insufficient.

Minute changes in temporal muscle thickness were detectable using the ultrasonographic machine M-Turbo, a portable ultrasonographic machine with regular resolution. Recently, increasing numbers of portable ultrasonographic machines with higher levels of resolution have become available, suggesting the utility of ultrasonography for nutritional monitoring in home-care settings. Further studies are needed to assess the feasibility and effectiveness of nutritional intervention programs guided by ultrasonographic evaluation of the temporal muscle.

We believe that this study will have a significant impact on the practice of nutritional care, because it describes a new method for nutritional monitoring that can detect energy inadequacy in older adults with impaired physical and cognitive functions. This method will be particularly useful in home-care settings because it is non-invasive, can be performed on patients in their beds, and does not require any active involvement of patients or caregivers. In clinical applications, this method can help clinicians to evaluate current nutritional care plans and take appropriate action to modify the plan as necessary: a nutritional intervention should be modified if the percentage change in temporal muscle thickness is less than − 3.6%, to meet the patient’s energy requirements. Thus, this newly developed nutritional monitoring method would assist older adult patients receiving home care by improving their nutritional status, leading to better physical and clinical outcomes.

There are several limitations to this study. First, the TEE was not measured directly, because the direct measurement of energy metabolism by means such as indirect calorimetry can cause a significant physical burden for bedridden elderly patients. We estimated TEE using the HB equation, which is widely accepted in today’s clinical practice in Japan. Although the HB equation has been reported to overestimate BEE [27, 28], the error of the estimation is considered to be consistent among individuals. Therefore, these estimation errors do not affect our result of a correlation between a change in temporal muscle thickness and energy adequacy. Also, we defined stress and activity factors in detail, based on previous evidence [17–26], to reduce random estimation errors. Second, there was a limited number of participants with severely impaired energy intake, so we could not determine a cutoff value to detect severe energy deficiency. In addition, our cohort did not include obese participants with excessive energy intake, which means the current results could not be generalized to obese bedridden individuals. Moreover, the data was not analyzed by sex due to the limited sample size. Given the previous report that male has a greater temporal muscle thickness than female [29], the data should be analyzed by sex in further study. Third, 13 participants (21.3%) were excluded from the analysis due to the poor quality of their ultrasonographic images. However, there were no significant differences in the basic characteristics between those who were excluded and those included in the analysis. Thus, this exclusion does not change the interpretation of our results.

## Conclusions

We demonstrated that percentage changes in temporal muscle thickness are significantly correlated with energy adequacy in bedridden older adults. A percentage change in temporal muscle thickness could detect moderate energy inadequacy (adequacy < 75%), with a cutoff value of − 3.6%. These results suggest that the ultrasonographic evaluation of temporal muscle thickness using a portable high-resolution ultrasonographic machine has the potential to be a useful nutritional monitoring method in older adults.

## Data Availability

The datasets used and/or analyzed during the current study are available from the corresponding author on reasonable request.

## Abbreviations

ADL: Activities of daily living
BMI: Body mass index
AC: Arm circumference
AMC: Arm muscle circumference
CC: Calf circumference
HB: The Harris–Benedict equation
BEE: Basal energy expenditure
TEE: Total energy expenditure
